# Antibiotic Prescribing in Dental Practice: A Cross-sectional Survey in Trinidad and Tobago

**DOI:** 10.1016/j.identj.2026.109588

**Published:** 2026-05-20

**Authors:** Francis Kalemeera, Ian Hosein, Sandeep Maharaj, Asha Ramkhalawan, Rajeev Peeyush Nagassar, Rahul Naidu

**Affiliations:** a Faculty of Medical Sciences, School of Pharmacy, The University of the West Indies, St. Augustine, Tunapuna-Piarco, Trinidad and Tobago; b Faculty of Medical Sciences, School of Medicine, The University of the West Indies, St. Augustine, Tunapuna-Piarco, Trinidad and Tobago; c Faculty of Medical Sciences, School of Dentistry, The University of the West Indies, St. Augustine, Tunapuna-Piarco, Trinidad and Tobago; d The Sangre Grande Hospital, Eastern Region Health Authority, Sangre-Grande, Trinidad and Tobago

**Keywords:** Antibiotics, Resistance, Dentists, Antimicrobial stewardship, Guidelines

## Abstract

**Background:**

Antimicrobial resistance (AMR) is a global threat. Trinidad and Tobago used the World Health Organisation’s Global Action Plan to develop its National Action Plan (NAP) against AMR. A review of the NAP showed the need for use-based studies to direct interventions against AMR. This study was designed to provide evidence on dental prescribing habits, to support interventions against the emergence of antibiotic-resistant strains.

**Methods:**

A cross-sectional study. A consent letter and questionnaire were developed in REDCap®, a link to which was sent to registered dentists. The questionnaire collected demographic information, knowledge of antibiotics and dental practice. SPSS version 22 was used for analysis: significance at a *P*-value <0.05 and the confidence interval at 95%.

**Findings:**

Seventy-two dentists participated. For penicillin-allergic patients, 38.6% (24/62) reported prescribing clindamycin, and 4.8% (3/62) reported prescribing amoxicillin. A total of 9.8% (6/61) avoided antibiotic prescription in pregnancy. Actions taken for patients already on antibiotics varied greatly – assess, discontinue, continue and switch. Satisfactory individual-level compliance with prophylaxis guidelines was observed in 29.8% (17/56), 47% (21/45), and 20% (9/48) of participants for dental procedures, prosthetic joints, and cardiac conditions, respectively. Satisfactory individual-level compliance with guidelines on the therapeutic use of antibiotics for dental infections was observed in 74.1% (40/54). Satisfactory group-level compliance with prophylaxis guidelines was observed in 8/19 dental procedures and 2/14 cardiac conditions. Satisfactory compliance with guidelines on the therapeutic prescription of antibiotics was observed in 6/10 dental infections. No variable predicted compliance.

**Conclusion:**

The variability in compliance highlights a need for improved education and awareness.

**Clinical relevance:**

may impact antimicrobial stewardship activities, including production of training materials and development of local treatment recommendations.

## Background

Antibiotic resistance – also referred to as antimicrobial resistance (AMR) – is a global threat associated with increased morbidity and mortality. It is predicted to account for approximately 10 million deaths per year by 2050 and is linked to excessive prescription and use of antibiotics.^1^, ^2^, ^3^ According to the World Health Organisation’s 2025 global antibiotic resistance surveillance report, AMR is the leading cause of death.^4^ The rising prevalence of AMR is complicated by a reduction in innovation of new antibiotics.^1^^,^^5^

Frequently, dentists have mechanically managed dental infections but also have used antibiotics therapeutically and prophylactically.^6^, ^7^, ^8^ Some dental infections are managed without systemic antibiotics; however, when prescribed, inappropriate use has been documented.^9^, ^10^, ^11^ Inappropriate use of antibiotics in Trinidad and Tobago’s dental practices has the potential to foster alterations of the microbiome, generation of antibiotic resistance and increased morbidity, mortality and costs of care. Trinidad and Tobago adapted the World Health Organisation’s global action plan on AMR.^12^ A 2019 review of the National Action Plan (NAP) against AMR, identified antibiotic-related use and consumption studies as strategic research areas to guide interventions.^13^ Nonetheless, a 2021 progress report on NAPs in the region of the Americas showed that most countries had regulations for antibiotics prescriptions, but that their execution may not be monitored or enforced. For Trinidad and Tobago, the monitoring of consumption of antibiotics was estimated at 26%.^14^ The consumption of antibiotics is positively related to the number of prescriptions, and dentists’ prescriptions account for a significant quantity.^15^^,^^16^ However, the proportion of antibiotic prescriptions issued by dentists that comply with guidelines is unknown. In addition, many studies are conducted in countries with different economic and health resources, thus presenting difficulties with extrapolation of findings. This calls for the implementation of local research.

Since dentists are key actors in the prescription of antibiotics, this study was designed to provide local evidence on dental antibiotic prescribing habits. The findings are intended to strengthen antibiotic stewardship-related interventions with a focus on dentists.

## Methods

### Study design

This was a cross-sectional study. It was principally quantitative in nature, but had a qualitative part, in which the participants provided reasons for the actions they reported taking.

### Sample characteristics

The target participants were dentists registered in Trinidad and Tobago. The estimated sample size was 244, using the OpenEpi calculator, based on a population of 600, a hypothesised frequency outcome factor in the population of 50% and confidence limits at 5%. Convenience sampling was used.

### Data collection tool

We developed a questionnaire for dentists in practice, which was adapted from the study by Mansour et al.^34^ and designed in REDCap® (The questionnaire is available in Appendix A). It comprised sections covering participants’ demographics, knowledge of antibiotics and dental practice in relation to prescription of antibiotics. The questionnaire queried the use of antibiotics in 4 categories: (1) prophylaxis for dental procedures; (2) the prescription of antibiotics for orodental infections; (3) antibiotic prophylaxis for special conditions (nondental and noncardiac); and (4) antibiotic prophylaxis for cardiac conditions. The cardiac conditions were subdivided into increased and not increased risk of infective endocarditis (IE). The increased risk category was further split into those that did and those that did not require special consideration for antibiotic prophylaxis against IE (special consideration is given to patients with prosthetic cardiac valves, previous IE, unrepaired cyanotic heart disease and atrial septal defect after months of repair). These categorisations were guided by the Faculty of General Dental Practice/Faculty of Dental Surgery (FGDP/FDS) guidelines^17^; the Implementation Advice by the Scottish Dental Clinical Effectiveness Programme^18^; and the National Institute for Health and Care Excellence (NICE) Guideline 64.^19^ The response was either ‘Yes’, ‘No’ or ‘Don’t know’ for all questions regarding the prescription of antibiotics.

### Survey administration

The participants received the link to the questionnaire via an email that was sent by the Dental Council of Trinidad and Tobago.

### Definition of compliance

The FGDP/FDS Guidelines was used to assess compliance. The guideline provided 3 antibiotics use scenarios: recommended; not usually recommended and not recommended. The response ‘Yes’ was correct for conditions or procedures where the guidelines recommended the prescription of antibiotics. The response ‘No’ was correct for conditions or procedures where the guidelines did not recommend the prescription of antibiotics. Conditions or procedures for which the guidelines stated that the prescription of antibiotics was ‘not usually recommended’, were deemed ‘not able to assess’. A response in agreement with the guidelines attracted 1 mark, otherwise no mark was given. A calculated compliance ≥75%, was deemed satisfactory, a level adopted from a study by Haridi et al.^20^

### Data analysis

Data were described using numbers and proportions of participants and related variables. Proportions were calculated for qualifications, places of work (private or public), age-groups, engagement in continuous professional development (CPD), the number of patients seen, the prescription of antibiotics and the factors that influenced antibiotic prescribing. The names and proportions of antibiotics that (1) replaced amoxicillin in penicillin allergic patients; (2) were prescribed for pregnant patients and for breastfeeding mothers, were tabulated. The number and proportion of factors that influenced prescription of antibiotics were documented. The reasons prescribers gave for their actions when handling patients already on antibiotics were divided into various assessment categories. The findings on assessment were documented and the corresponding actions were recorded.

For each category, compliance was assessed at the individual-level and at the group-level. For individual-level compliance we divided the number of correct responses (dividend) by the number of conditions or procedures (divider) per category. The resultant fractions (quotient) were converted into percentages, which represented the level of compliance. For group-level compliance we divided the number of correct responses (dividend) per condition or procedure by the number of participants (divider). The resultant fractions (quotient) were converted into percentages, which represented the level of compliance.

We conducted binary logistic regression analysis. The outcome variable was individual compliance, with a compliance level ≥75% deemed satisfactory, while below 75% was deemed unsatisfactory. Univariate regression analysis was conducted to assess the relationship between the potentially predictor variables and the outcome variable. Then these potentially predictor variables were included in a multivariate logistic regression model to identify the predictors of compliance. Significance was set at a *P*-value of ≤.05 and the confidence interval at 95%. The statistics were conducted using SPSS version 22.

### Ethics

Ethics: This study commenced following approval by the Campus Research and Ethics Committee (CREC). The data were received within REDCap® and were downloaded into Excel. The participants consented and were anonymised. The reference code of the approval is CREC – SA.3130.02/2025. The study followed the Declaration of Helsinki.

## Findings

### Participant characteristics

Characteristics were collected for up to 72 dental practitioners. Most (91.7%, *n* = 66) were general practitioners, 68.1% (*n* = 59) held bachelor’s or master’s degrees and 9.7% (*n* = 6) had specialties. Many (81%, *n* = 58) worked in private clinics and 41.7% (*n* = 30) were in the 30 to 40 years age group. Only 18.1% (*n* = 13) engaged in CPD, though 77.8% (*n* = 56) reported to have read journal articles in the past 5 years and less than 50% attended conferences. Most (69.4%, *n* = 50) saw fewer than 41 patients weekly. About 15.3% (*n* = 11) reported that 26% to 50% of consultations involved antibiotic prescriptions and 24.1% (*n* = 14) faced barriers to guideline compliance. Importantly, almost 50% of reported patient-induced pressure to prescribe and some were influenced by the presence of antibiotic samples, drug representatives and socioeconomic status of the patient (Table 1).

**Table 1:** Characteristics of the participants and factors associated with their work.

Variable	*n* (%)	
Number of specialties: *n* (%)	None (general practitioners)	66 (91.7)
	2	5 (6.9)
	7	1 (1.4)
	*n*	72
Qualification level	BDS	38 (52.8)
	Diploma	11 (15.3)
	Masters	21 (29.2)
	PhD	2 (2.7)
	*n*	72
Age group (y)	<30	1 (1.4)
	30-40	30 (41.7)
	41-50	21 (29.2)
	51-60	16 (22.2)
	>60	4 (5.5)
	*n*	72
Settings	Private clinic	58 (80.6)
	Public hospital and private clinic	5 (6.9)
	Mixed facility	8 (11.1)
	Private clin and mixed facility	1 (1.4)
	*n*	72
Activities to keep abreast with information	None	6 (8.3)
	National conferences	29 (40.3)
	International conferences	24 (33.3)
	Continuous professional development	13 (18.1)
	Lectures (past 5 y)	32 (44.4)
	Article reading (past 5 y)	56 (77.8)
Patients seen per wk	≤20	21 (29.6)
	21-40	29 (40.9)
	41-60	14 (19.7)
	61-80	4 (5.6)
	>80	3 (4.2)
	*n*	71
Frequency of consultations involving the prescribing of antibiotics	10% or less	38 (52.8)
	25% or less	21 (29.2)
	50% or less	11 (15.2)
	More than 75%	1 (13.8)
	*n*	72
Experiencing blocks to comply with guidelines	Yes	14 (24.1)
	No	44 (75.9)
	*n*	58
Antibiotic prescribed for penicillin allergic patients	Erythromycin	8 (12.8)
	Clindamycin	24 (38.6)
	Metronidazole	14 (22.6)
	Clarithromycin	4 (6.9)
	Ciprofloxacin	1 (1.5)
	Azithromycin	8 (12.8)
	Amoxicillin	3 (4.8)
	*n*	62
Antibiotics prescribed in pregnancy	Amoxicillin	25 (41.0)
	Amoxicillin/clavulanic acid	18 (29.5)
	Penicillin (unspecified)	7 (11.5)
	Cefuroxime	1 (1.6)
	Azithromycin or metronidazole	2 (3.3)
	None	6 (9.8)
	Consult	2 (3.3)
	*n*	61
Antibiotics prescribed in breastfeeding mothers	Amoxicillin	26 (41.9)
	Amoxicillin/clavulanic acid	19 (30.7)
	Penicillin (unspecified)	9 (14.5)
	Cefuroxime	1 (1.6)
	None	6 (9.7)
	Consult	1 (1.6)
	*n*	62
Consultation of physician for cardiac patients	Never	1 (1.6)
	Sometimes	27 (43.6)
	Always	34 (54.8)
	*n*	62
Action for patient already taking antibiotics	Continue antibiotic course	25 (40.3)
	Action depends on target of antibiotic therapy	37 (59.7)
	Change antibiotic	1 (1.6)
	Action depends on time (change if antibiotic taken during last month)	13 (21.0)
	Discontinue antibiotic course	1 (1.6)
	Continue antibiotic course and add vitamins	–
	Increase the dose	–
Feeling pressure to prescribe antibiotics	Never	30 (50.9)
	Sometimes	27 (45.7)
	Often	1 (1.7)
	Always	1 (1.7)
	*n*	59
Factors influencing the prescribing of antibiotics	Previous antibiotic experience	53 (39.0)
	Comorbidities of the patient	51 (86.4)
	Socio-economic status of the patient	29 (49.2)
	Price of the antibiotic	27 (45.8)
	Samples availability	5 (8.5)
	Medical representatives	3 (5.1)

All variables are presented by numbers and percentages. The percentages are presented in brackets.

Seven antibiotics were mentioned as replacements for a penicillin in allergic patients (Table 1). Of these, clindamycin dominated, followed by metronidazole. Ciprofloxacin and amoxicillin were also listed (Table 1). During pregnancy, 73% (*n* = 45) prescribed amoxicillin, while 9.8% (*n* = 6) avoided antibiotics. For breastfeeding, 89.6% (*n* = 26 of 29 participants) favoured amoxicillin.

**Fig. 1:**
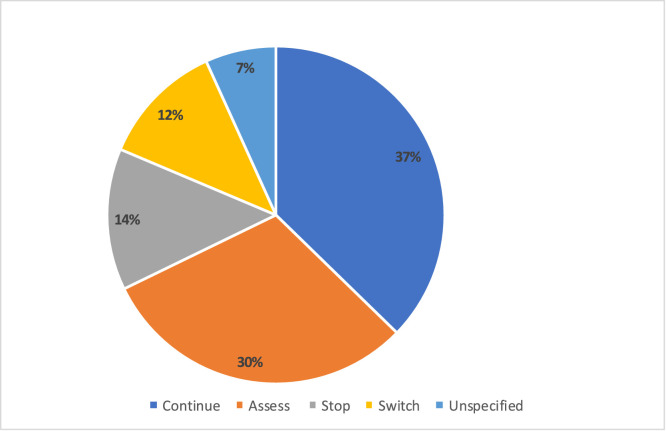
How to manage antibiotic therapy in patients already taking antibiotics.

Nearly half (49.2%, *n* = 29) of the 59 participants who responded to the question, felt patient pressure to prescribe antibiotics. Other influencing factors included previous antibiotic experience (89.8%, *n* = 53) and comorbidities (86.4%, *n* = 51), (Table 1). For patients already taking antibiotics 37.3% (22/59) would continue the antibiotic course (Figure 1). A variety of suboptimal actions were suggested, including switching antibiotics for fungal superinfection, discontinuing antibiotics for lack of effectiveness, continuing antibiotics if they were prescribed and stopping antibiotics if they were obtained without a prescription (Table 2).

**Table 2:** Issues assessed for patients already taking antibiotics, the findings on assessment and the actions taken.

		Actions					
Assessment category	Findings	1	2	3	4	5	6
Coverage	1. Insufficient	Add for coverage	Discontinue	Change			
	2. Sufficient	Continue					
Outcome	1. Target not achieved	Increase dose	Change antibiotic	Consider intravenous administration	Discontinue antibiotic	Continue antibiotic	Request culture and sensitivity test
	2. Fungal superinfection infection	Change antibiotic					
	3. Allergic reaction	Change antibiotic					
Dose	1. Low	Increase dose					
	2. Appropriate	Continue antibiotic					
Health condition	1. Antibiotic not needed	Discontinue antibiotic					
	2. Depth of infection	No action recorded					
Prescription status	1. No prescription (self-care)	Discontinue if the antibiotic is taken incorrectly					
	2. Prescription present	Continue antibiotic					
Antibiotic use history	1. 2 wk	Change antibiotic					
	2. 4 wk	Change antibiotic					
Cost	1. High for patient	Change antibiotic					
Drug interaction	1. Drug interaction present	Change antibiotic					
Concern for resistance	N/A	Continue to avoid resistance					
Length of use	1. Prolonged administration	No action recorded					
	2. Within normal schedule	No action recorded					

Column 1: aspects assessed for patients already taking antibiotics. Column 2: findings on assessment. Next: actions taken.

### Antibiotic prophylaxis for dental procedures

Compliance with prophylactic use was evaluated for 19 dental procedures and up to 58 participants responded to this section of the questionnaire (Supplementary Material, Table 1). Of the 19 dental procedures, 10 were invasive, 5 were minimally invasive and 4 were noninvasive. The FGDP/FDS guidelines recommended antibiotic prophylaxis in only 2 of the 19 procedures, namely: bone graft and flap surgery, both of which are invasive in nature. For both, the compliance level was unsatisfactory: 57% and 67%, respectively. Of the remaining 17 dental procedures, for which antibiotics were not recommended, satisfactory compliance (≥75%) was reported in 8: braces (97%), crowns (97%), simple extractions (91%), intraligamentary local anaesthesia (93%), local anaesthesia (93%), prosthesis (93%), restoration (93%) and scaling (93%). Unsatisfactory compliance in the remaining 9 dental procedures, for which antibiotics were not recommended, was averaged at 44.4% (range: 11%-62%). Examples include: tumour resection (11%), implant (24%) and maxillary extraction (62%). Only 29.8% (17/57) of the participants were satisfactorily compliant across all the above-mentioned dental procedures. Details are documented in the Supplementary Material’s Table 1 that is titled, Compliance with guidelines on prophylactic use of antibiotics in dental procedures.

### Antibiotic therapy for dental infections

Compliance with therapeutic use of antibiotics was assessable for 10 of the 15 dental infections and up to 54 participants responded to this part of the questionnaire (Supplementary Material, Table 2). The FGDP/FDS guidelines recommended antibiotics therapy for 5 of the 10 dental infections. Satisfactory compliance (≥75%) was observed in 4 of the 5 dental infections, for which antibiotic therapy was recommended, namely: aggressive periodontitis (81%), cellulitis (98%), osteomyelitis (91%) and salivary gland infection (80%). Unsatisfactory compliance was observed for fistula (65%). For the 5 dental infections, in which antibiotic therapy was not recommended, satisfactory compliance was noted for gingivitis (91%) and tooth decay (96%). Unsatisfactory compliance was observed for the last 3 infections, namely: peri-implantitis (7%), pulpitis (57%) and chronic periodontitis (61%). Overall, 74.1% (40/54) were compliant across assessable conditions. More information, including the 5 dental infections not mentioned above, is presented in the Supplementary Material’s Table 2 that is titled, Compliance with guidelines on use of antibiotics therapeutically for dental infections.

### Antibiotic prophylaxis for special medical conditions

Of the 9 nondental, noncardiac conditions, only prosthetic joints were assessable for compliance (Supplementary Material, Table 3). Forty-five participants responded to this area of the questionnaire. Twenty-one (*n* = 21) participants of the 45 correctly agreed that antibiotic prophylaxis is not recommended in patients with prosthetic joints. The details, including all the nondental and noncardiac conditions, are presented in the Supplementary Material’s Table 3, which is titled, Compliance with guidelines on the prophylactic use of antibiotics in special medical conditions.

### Antibiotic prophylaxis for cardiac conditions

**Fig. 2:**
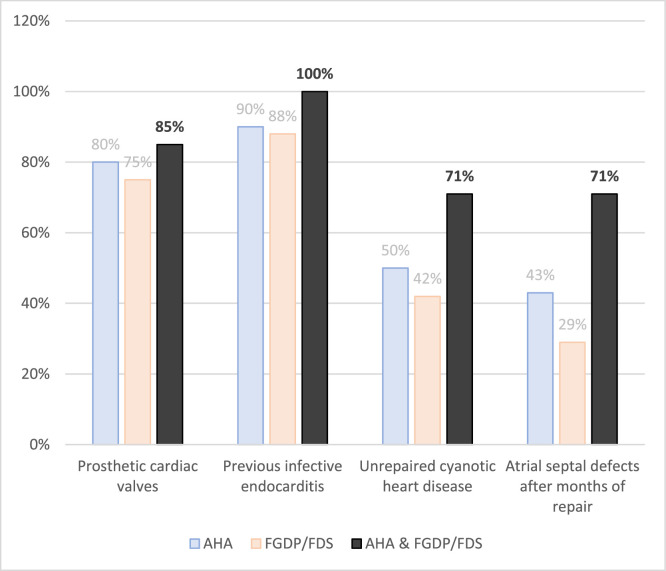
AP compliance in patients at high-risk for infective endocarditis. The figure shows the percentage of participants who were compliant according to the guidelines they possessed.

An assessment on whether antibiotic prophylaxis was required for 14 cardiac conditions was conducted (Supplementary Material, Table 4) and 48 participants responded to this part of the questionnaire. The FGDP/FDS guidelines recommended antibiotics prophylaxis for 4 of the 14 cardiac conditions. Satisfactory compliance was notable for 2 out of the 4 conditions for which antibiotic prophylaxis was recommended, namely: prosthetic cardiac valves (79%) and previous IE (90%). For the other 2 unsatisfactory compliance was documented: atrial septal defect after months of repair (33%) and unrepaired cyanotic heart disease (48%). For conditions where antibiotics were not recommended, average compliance was 41.9% (range: 10%-71%). Examples include: rheumatic heart disease (21%), mitral valve prolapses with regurgitation (21%) and cardiac transplantation recipients (10%). Only 20% (9/48) were fully compliant for cardiac-related prophylaxis. Participants possessing both the American Heart Association (AHA) and the FGDP/FDS guidelines, tended to have higher compliance levels for prevention of IE in high-risk patients (Figure 2). More information, including the 14 cardiac conditions, is available in the Supplementary Material’s Table 4 that is titled, Compliance with guidelines on the prophylactic use of antibiotics before dental procedures in patients with cardiac conditions.

### Regression analysis for predictors of compliance

Regression analysis: Univariate results showed a 1-year increase in practice associated with a 10% increase in compliance (Odds Ratio [OR] = 1.1; 95%, confidence interval [CI]: 1.0-1.2; *P* = .068), not statistically significant. Each 10-year increase in age associated with a 130% increase in compliance (OR = 2.3; 95% CI: 1.0-5.2; *P* = .05), also not statistically significant. Dentists in public/mixed settings associated with a nonstatistically significant 50% increase in compliance (OR = 1.5; 95% CI: 0.7-3.5; *P* = .333). No significant associations for specialty (OR = 0.9; 95% CI: 0.3-2.6; *P* = .778) or qualification level (OR = 0.7; 95% CI: 0.3-1.5; *P* = .334) were noted. Multivariate analysis did not identify any predictors of compliance.

## Discussion

This study reveals gaps in knowledge and variable practice regarding antibiotics in pregnancy, penicillin-allergic patients and patients already taking antibiotics. Variations were evident in prophylactic and therapeutic antibiotic use, including antibiotic prophylaxis (AP) for dental procedures with and without cardiac conditions, antibiotic therapy for orodental infections and prophylaxis for patients with prosthetic joints. Susceptibility to nonmedical inducements to prescribe antibiotics was noted. No single variable predicted compliance with guidelines.

The frequent prescription of amoxicillin aligns with other studies.^21^, ^22^, ^23^Alternatives for penicillin-allergic patients reported were similar in class to those reported by Juarez-Membreno et al.^24^ although some participants reported prescribing amoxicillin to penicillin-allergic patients – a significant knowledge gap, possibly linked to low CPD engagement. Insufficient knowledge on the management of allergies was reported by Golchini et al. who recommended CPD and wider dissemination of international guidelines as interventions.^25^ When selecting alternatives, the spectrum of activity must be considered, as amoxicillin shares the spectrum of activity with macrolides and, to a lesser extent, clindamycin, making these alternatives reasonable for penicillin-allergic patients.^26^ Metronidazole targets anaerobes and is not a complete substitute; it is sometimes combined with amoxicillin to broaden coverage.^27^, ^28^, ^29^, ^30^ Some participants precluded the prescription of antibiotics in pregnancy, thus exposing gaps in knowledge on the use of antibiotics. These findings create opportunities to strengthen antibiotic stewardship via the production of local guidelines and training.

Prescribing antibiotics in response to patient pressure is not unique to dentists in Trinidad and Tobago. In a USA-based study, Huynh et al. found that dentists succumbed to pressure demanding the prescription of antibiotics from patients and from other health care professionals.^31^ Patient-induced pressure on dentists to prescribe antibiotics was documented by Khatib et al. and Membre et al.^32^^,^^33^ Huynh et al. reiterate that submission to such demands may be associated with limited awareness of the association between inappropriate use of antibiotics and resistant bacterial organisms and insufficient knowledge of adverse drug reactions that may result.^31^

Regarding patients already on antibiotics, 37.3% of the participants supported continuation; other actions varied (assess, continue, stop, switch) – comparable but somewhat lower than what was documented by Mansour et al.^34^ To switch from an antibiotic if the same had been taken 2 to 4 weeks before the current therapy is recommended in the management of aggressive periodontitis, due to an increased risk for antibiotic resistance^35^; however, we could not confirm that the participants response was associated with the management of aggressive periodontitis. The variety of nonoptimal actions, when handling these patients, such as discontinuing treatment when the target of therapy is not achieved and when the drug was obtained through self-care from a pharmacy – that is, without a prescription – and the dose administered is incorrect, reflects inconsistent knowledge, exposing the need for continuing professional development courses on antibiotic therapy. In Bohmer et al.’s study, the dentists reported a lack of empowerment for making therapeutic decisions, thus interfering with guideline conformity.^36^

Historically, antibiotic prophylaxis for invasive dental procedures became common to prevent bacteraemia-associated IE. However, newer evidence showed that bacteraemia was relatively common after routine oral hygiene, although at lower frequencies than those estimated following invasive dental procedures.^37^^,^^38^ AHA and European Society of Cardiology (ESC) guidelines were changed in response, now recommending AP for high-risk patients – that is, patients with a history of IE, prosthetic cardiac valves, congenital cyanotic heart disease, prosthetic material used in valve repair, whereas NICE advises against routine AP, creating guideline divergence and confusion.^39^, ^40^, ^41^ The purpose of AP in these high-risk patients is to reduce the intensity and duration of bacteraemia.^37^ Participants with both AHA and FGDP/FDS guidelines reported better compliance for high-risk conditions, suggesting guideline access/use matters.

AHA, ESC and NICE do not recommend AP for moderate risk patients (eg, prior rheumatic fever, bicuspid aortic valve prolapse, mitral valve prolapse, calcific aortic stenosis and unrepaired congenital valve anomalies).^17^ Low compliance for these conditions was observed, possibly relating to limited CPD engagement. Some participants never consulted cardiologists, which may risk patient safety.

Our study had some limitations. The low response rate and partial responses limit generalisability. Though not extrapolatable, the descriptive statistics reflect respondent practices. However, self-reported practice may not be a true reflection of the actual practice. Therefore, the true compliance may be lower than estimated. Some procedures could not be assessed for compliance because AP was situational: required in some, but not in other similar cases. The FGDP/FDS Good Practice Guidelines (2020) were used for judgements; variations between guidelines exist, but responses for high-risk patients were similar across guideline types.

## Conclusion

High compliance for routine dental procedures suggests familiarity with standard practice, but low compliance in complex scenarios, such as cardiac conditions and invasive procedures, indicates confusion or lack of guideline dissemination. Our study has identified knowledge gaps in the selection of antibiotics in penicillin-allergic patients, pregnant patients and in the management of patients already taking antibiotics. There is therefore a need for continuing professional education on antibiotic prescription in dentistry.

## Author contributions

*Concept design*: Kalemeera, Hosein, Maharaj and Naidu. *Data acquisition*: Kalemeera and Ramkhalawan. *Data analysis*: Kalemeera, Hosein, Maharaj, Ramkhalawan and Nagassar. *Drafting of the manuscript*: Kalemeera, Hosein, Maharaj, Ramkhalawan, Naidu and Nagassar. *Article completion/approval*: Kalemeera, Hosein, Maharaj and Naidu.

## Conflict of interest

None disclosed

## Appendix Supplementary materials

Image, application 1

Image, application 2

## Footnotes

Supplementary material associated with this article can be found in the online version at doi:10.1016/j.identj.2026.109588.
