# Supplementary material for: Antibiotic Prescribing in Dental Practice: A Cross-sectional Survey in Trinidad and Tobago
Source: Int Dent J. 2026 May 20;76(4):109588. doi: 10.1016/j.identj.2026.109588 (PMC13213650; doi:10.1016/j.identj.2026.109588)
Supplement: Supplementary file 1 [file mmc1.pdf]

Antimicrobial Prescribing in Dentistry

Dear Doctor,

Re: Consent request letter

We are inviting you to participate in this study, titled Antimicrobial Prescribing in Dentistry.

Through this study, we seek to describe how antibiotics are prescribed or potentially prescribed prophylactically prior to or after dental procedures to prevent local or systemic infections. Further, via this study we intend to describe how antibiotics are prescribed for the management of specific orofacial/dental infections. Furthermore, we intend to find out if there is a relationship between the prescription of antibiotics and a number of factors for example, the type of procedure, and patient characteristics. The penultimate goal of the study is to develop practicable strategies that can be used to strengthen appropriate antimicrobial prescribing in dentistry, if the study reveals the need thereof. The ultimate goal of this study will be the appropriate use of antibiotics, and the eventual contribution towards reduction in the emergence of antibiotic-resistant micro-organisms. The findings of this study will be published in local and international forums such as scientific journals, conferences, etc.

Your privacy will be preserved, and confidentiality strictly observed. This is ensured by NOT collecting identification data. Additionally, the collected data will be stored in a system (REDCap) to which only the principle investigator has access. Scrutiny will be applied to the data analysis findings to completely eliminate unintential breach of confidentiality. While there is no direct benefit for you as a participant, we envisage, as mentioned above, positive outcomes of the research.

You are eligible to participate in this study if you are registered with the dentistry board of Trinidad and Tobago; and are currently practicing dentistry; stopped active practice not more than six months ago.

By this letter, you are requested to participate in this study. You have the right to accept or reject participation in this study.

I have read the consent request letter, and...

- ◯ I consent participating in the study
- ◯ I deny consent to participate in the study

PARTICIPANT CHARACTERISTICS

1 In which region are you practicing dentistry? OR If not working now, in which region did you practice dentistry within the past six months?

- ☐ Couva/Tabaquite/Talparo
- ☐ Diego Martin
- ☐ Mayaro/Rio Claro
- ☐ Penal/Debe
- ☐ Princes Town
- ☐ Sangre Grande
- ☐ San Juan/Laventille
- ☐ Siparia
- ☐ Tunapuna/Piarco

2 Select your gender

- ◯ Male
- ◯ Female

Select the age-group you belong to

- ◯ < 30 years
- ◯ 30 - 40 years
- ◯ 41 - 50 years
- ◯ 51 - 60 years
- ◯ >60 years

How MANY YEARS have you been in active dental practice?

____

From the list provided, select your areas of specialty

- ☐ General practitioner
- ☐ Oral surgeon
- ☐ Endodontic
- ☐ Implant surgeon
- ☐ Pediatric dentist
- ☐ Orthodontist
- ☐ Restorative Dentist
- ☐ Prosthodontics
- ☐ Periodontics
- ☐ Other

What is the level of your post-graduate qualification, if any?

- ☐ None
- ☐ Master's degree
- ☐ University Diploma
- ☐ PhD

Where do you obtain your post-graduate qualifications from?

- ◯ Trinidad and Tobago
- ◯ Not from Trinidad and Tobago
- ◯ Not applicable

What kind of setting(s) do you work in?

- ☐ Private clinic
- ☐ Private Hospital
- ☐ Public hospital
- ☐ Mixed public and private
- ☐ Not Applicable

What sources of information do you use for continuing professional development?

- ☐ None
- ☐ National conferences
- ☐ International conferences
- ☐ Continuing education lectures

Do you use any guidelines when prescribing antibiotics for prophylaxis against endocarditis in susceptible patients?

- ◯ Yes
- ◯ No

Which guidelines do you use?	☐ Do not know<br>☐ Guidelines provided during dental qualification years<br>☐ American Heart Association (AHA)<br>☐ Antimicrobial Chemotherapy (BSAC)<br>☐ Agence Française de Sécurité Sanitaire des Produits de Santé (Afssaps)<br>☐ National Institute for Health and Care Excellence (NICE) and the British Society for Dentistry<br>☐ Guidelines published in Trinidad and Tobago<br>☐ Not applicable
Have you attended any lectures relating to the use of antibiotics in dental medicine during the past 5 years?	◯ Yes<br>◯ No
Have you read any journal article relating to the use of antibiotics in dental medicine during the past 5 years?	◯ Yes<br>◯ No
On average, how many patients do you see per week?	__________________________________
Approximately, what percentage of your dental consultations involve prescription of antibiotics?	◯ 10% or less<br>◯ 25% or less<br>◯ 50% or less<br>◯ 75% or less<br>◯ More than 75%

ATTITUDE / PERCEPTION

Do you encounter any blocks/hinderance to complying with guidlines?

- ◯ Yes
- ◯ No

Which antibiotic do you commonly prescribe instead of a penicillin, because the patient is allergic to penicillin?

◯ Erythromycin ◯ Clindamycin ◯ Ceftazidime ◯ Other

Other Antibiotic

____

Which antibiotic do you commonly prescribe for dental patients who are pregnant?

____

Which antibiotic do you commonly prescribe for breastfeeding mothers?

____

How often do you seek advice from a physician when managing dental patients with cardiac illnesses?

◯ Never ◯ Sometimes ◯ Always ◯ Not noticed or seen such patients

What would you do if the dental patient is already taking antibiotics?

☐ Continue antibiotic course ☐ Action depends on the target of antibiotic therapy ☐ Change the antibiotic ☐ Action depends on time (change if antibiotic taken during last month) ☐ Discontinue antibiotic course ☐ Continue antibiotic course and add vitamins ☐ Increase the dose

Very briefly state the reason(s) for the choice(s) you have made regarding what you would do for a dental patient who is already receivng antibiotics

____

To what extent do you feel pressure from patients to prescribe antibiotics?

- ◯ Never
- ◯ Sometimes
- ◯ Often
- ◯ Always

Which of these factors influence the prescribing of antibiotics?

- ☐ Previous antibiotic experience
- ☐ Comorbidities of the patient
- ☐ Socio-economic status of the patient
- ☐ Price of the antibiotic
- ☐ Samples availability
- ☐ Medical representative visits

DENTAL PRACTICE - PART ONE

You are requested to indicate whether you prescribe or recommend prescription of PROPHYLACTIC antibiotics after these procedures.

	Yes	No	Don't know
Bone graft	◯	◯	◯
Braces	◯	◯	◯
Crown	◯	◯	◯
Crown lengthening	◯	◯	◯
Extraction: mandibular	◯	◯	◯
Extraction: maxillary	◯	◯	◯
Extraction: simple	◯	◯	◯
Flap surgery	◯	◯	◯
Frenectomy	◯	◯	◯
Germectomy	◯	◯	◯
Gingivectomy	◯	◯	◯
Implant	◯	◯	◯
Intraligamentary local anaesthesia	◯	◯	◯
Local anaesthesia	◯	◯	◯
Necrotic tooth	◯	◯	◯
Prosthesis	◯	◯	◯
Restoration	◯	◯	◯
Scaling	◯	◯	◯
Tumor resection	◯	◯	◯

DENTAL PRACTICE - PART TWO

Indicate with a YES or NO or DON'T KNOW whether you prescribe or recommend prescription of antibiotics to TREAT the following dental infections.

	Yes	No	Don't Know
Aggressive periodontitis	○	○	○
Apical abscess	○	○	○
Bacterial stomatitis	○	○	○
Cellulitis	○	○	○
Chronic periodontitis	○	○	○
Fistula	○	○	○
Gingivitis	○	○	○
Maxillary sinusitis	○	○	○
Osteomyelitis	○	○	○
Periapical abscess	○	○	○
Perimplantitis	○	○	○
Periodontal abscess	○	○	○
Pulpitis	○	○	○
Salivary gland infection	○	○	○
Tooth decay	○	○	○

ANTIBIOTICS USE FOLLOWING DENTAL PROCEDURES - PART ONE

Would you use antibiotics following dental procedures in patients with Cardiac Diseases

	Yes	No	Don't know
Prosthetic cardiac valves	○	○	○
Rheumatic heart disease	○	○	○
Mitral valve prolapse with valvular regurgitation	○	○	○
Previous infective endocarditis	○	○	○
Hypertrophic cardiomyopathy	○	○	○
Intravascular cardiac pacemakers	○	○	○
Myocardial infarction in the last six months	○	○	○
Cardiac transplantation recipients who develop cardica valvulopathy	○	○	○
Unrepaired cyanotic heart disease	○	○	○
Recently placed coronary stents	○	○	○
Atrial septal defects after months of repair	○	○	○
Ventricular septal defect with repair	○	○	○
Patent ductus arteriosus	○	○	○
Cardiac catheterization without stents	○	○	○

ANTIBIOTICS USE FOLLOWING DENTAL PROCEDURES - PART TWO

Would you use antibiotics following dental procedures in patients with non cardiac potentially high-risk diseases such as HIV and diabetes mellitus

	Yes	No	Don't know
HIV infection	○	○	○
Neutropenia	○	○	○
Cancer chemotherapy	○	○	○
Diabetes	○	○	○
Haematopoeitic stem cell or solid organ transplantation	○	○	○
Bisphosphonate therapy	○	○	○
Chronic steroid usage	○	○	○
Asplenism or status post splenectomy	○	○	○
Prosthetic joints	○	○	○

COMMENTS ON THE QUESTIONNAIRE & THE ENTIRE PROCESS

Should the questionnaire be modified? If yes, what changes do you suggest? __________________________________

Install Date __________________________________

Install Date (UTC) __________________________________

Participant Timezone __________________________________

Participant Code __________________________________
