# Supplementary material for: Antibiotic Prescribing in Dental Practice: A Cross-sectional Survey in Trinidad and Tobago
Source: Int Dent J. 2026 May 20;76(4):109588. doi: 10.1016/j.identj.2026.109588 (PMC13213650; doi:10.1016/j.identj.2026.109588)
Supplement: Supplementary file 2 [file mmc2.docx]

**Supplementary Material**

**Table One: Compliance with guidelines (GLs) on prophylactic use of antibiotics in dental procedures**

| **Dental Procedure** | **No. of participants** | **Should prophylactic antibiotics be given?** | | | | | **Compliance with GLs** |
| --- | --- | --- | --- | --- | --- | --- | --- |
|  |  | **Responses** | | | **Guidelines** | |  |
|  |  | **Yes** | **No** | **Don't know** | **Yes** | **No** |  |
| ^1^Bone graft | 58 | 34 | 7 | 17 | Yes | - | 57% |
| ^2^Braces | 58 |  | 56 | 2 | - | No | 97% |
| ^3^Crown | 58 | 1 | 56 | 1 | - | No | 97% |
| ^3^Crown lengthening | 56 | 21 | 29 | 6 | - | No | 38% |
| ^1^Extraction: mandibular | 55 | 19 | 34 | 2 | - | No | 62% |
| ^1^Extraction: maxillary | 55 | 19 | 34 | 2 | - | No | 62% |
| ^1^Extraction: Simple | 55 | 5 | 50 | - | - | No | 91% |
| ^1^Flap surgery | 55 | 37 | 14 | 4 | Yes | - | 67% |
| ^2^Frenectomy | 55 | 16 | 27 | 12 | - | No | 49% |
| ^1^Germectomy | 55 | 13 | 22 | 20 | - | No | 40% |
| ^2^Gingivectomy | 55 | 16 | 30 | 9 | - | No | 55% |
| ^1^Implant | 55 | 29 | 13 | 13 | - | No | 24% |
| ^2^Intraligamentary local anaesthesia | 56 | 1 | 52 | 3 | - | No | 93% |
| ^1^Local anaesthesia | 56 | 1 | 52 | 3 | - | No | 93% |
| ^1^Necrotic tooth | 56 | 23 | 33 | - | - | No | 59% |
| ^2,3^Prosthesis | 56 | 1 | 52 | 3 | - | No | 93% |
| ^1,3^Restoration | 56 | 1 | 52 | 3 | - | No | 93% |
| ^3^Scaling | 56 | 3 | 52 | 1 | - | No | 93% |
| ^1^Tumour resection | 55 | 37 | 6 | 12 | - | No | 11% |

**Table One Legend:** The superscript numbers explained: 1=invasive; 2=minimally invasive; 3=non-invasive. Columns six and seven indicate what the FGDP/FDS guidelines say: yes = prophylactic antibiotics recommended; No = prophylactic antibiotics not recommended. The last column shows the percentage of participants whose responses agreed with the guidelines.

**Table Two: Compliance with guidelines on use of antibiotics therapeutically for dental infections**

| **Condition** | **No. of participants** | **Should antibiotic therapy be given?** | | | | | | **Compliance with GLs** |
| --- | --- | --- | --- | --- | --- | --- | --- | --- |
|  |  | **Responses** | | | **Guidelines** | | |  |
|  |  | **Yes** | **No** | **Don't know** | **Yes** | **No** | |  |
| Aggressive periodontitis | 54 | 44 | 7 | 3 | Yes | - | | 81% |
| Apical abscess | 54 | 44 | 10 |  | Not able to assess | | | - |
| Bacterial stomatitis | 53 | 32 | 12 | 9 | Not able to assess | | | - |
| Cellulitis | 54 | 53 | 1 |  | Yes |  | | 98% |
| Chronic periodontitis | 54 | 21 | 33 |  | - | No | | 61% |
| Fistula | 54 | 35 | 19 |  | Yes |  | | 65% |
| Gingivitis | 54 | 4 | 49 | 1 | - | No | | 91% |
| Maxillary sinusitis | 54 | 38 | 13 | 3 | Not able to assess | | | - |
| Osteomyelitis | 54 | 49 |  | 5 | Yes | - | | 91% |
| Periapical abscess | 54 | 43 | 11 |  | Not able to assess | | | - |
| Periimplantitis | 54 | 41 | 4 | 9 | - | No | | 7% |
| Periodontal abscess | 53 | 46 | 6 | 1 | Not able to assess | | | - |
| Pulpitis | 54 | 23 | 31 |  | - | No | | 57% |
| Salivary gland infection | 54 | 43 | 2 | 9 | Yes | - | | 80% |
| Tooth decay | 54 | 1 | 52 | 1 | - | | No | 96% |

**Table Two Legend:** The first column consists of dental infections. Column six and seven indicate what the FGDP/FDS guidelines say: yes = antibiotics recommended; No = antibiotics not recommended. The last column (far right) shows the percentage of participants whose response agreed with the guidelines.

**Table Three: Compliance with guidelines on the prophylactic use of antibiotics in special medical conditions**

| **Special condition** | **Participants**  **No. of participants** | **Should antibiotics be given prophylactically?** | | | | | | **Compliance with GLs** |
| --- | --- | --- | --- | --- | --- | --- | --- | --- |
|  |  | **Responses** | | | **Guidelines** | | |  |
|  |  | **Yes** | **No** | **Don't know** | **Yes** | | **No** |  |
| HIV infection | 45 | 14 | 27 | 4 | Not able to assess | | | - |
| Neutropenia | 45 | 16 | 14 | 15 | Not able to assess | | | - |
| Cancer chemotherapy | 45 | 17 | 19 | 9 | Not able to assess | | | - |
| Diabetes | 45 | 20 | 24 | 1 | Not able to assess | | | - |
| Haematopoietic stem cell or solid organ transplantation | 45 | 20 | 9 | 16 | Not able to assess | | | - |
| Bisphosphonate therapy | 45 | 22 | 15 | 8 | Not able to assess | | | - |
| Chronic steroid usage | 45 | 18 | 18 | 9 | Not able to assess | | | - |
| Asplenism or status post splenectomy | 45 | 9 | 16 | 20 | Not able to assess | | | - |
| Prosthetic joints | 45 | 19 | 21 | 5 | - | No | | 47% |

**Table Three Legend:** The fifth column consists of the number of dentists who reported not knowing what to do. Column six and seven indicate what the FGDP/FDS guidelines say: yes = antibiotics recommended; No = antibiotics not recommended. The last column (far right) shows the percentage of participants whose response agreed with the guidelines.

**Table Four: Compliance with guidelines on the prophylactic use of antibiotics before dental procedures in patients with cardiac conditions.**

| **Cardiac conditions** | **No. of participants** | **Should prophylactic antibiotics be given?** | | | | | **Agreement with GLs (NICE)** |
| --- | --- | --- | --- | --- | --- | --- | --- |
|  |  | **Responses** | | | **Guidelines** | |  |
|  |  | **Yes** | **No** | **Don't know** | **Yes** | **No** |  |
| ^1^Prosthetic cardiac valves^a^ | 48 | 38 | 7 | 3 | Yes^b^ | - | 79% |
| Rheumatic heart disease | 48 | 36 | 10 | 2 | - | No | 21% |
| ^1^Mitral valve prolapsed with valvular regurgitation | 48 | 32 | 10 | 6 | - | No | 21% |
| ^1^Previous infective endocarditis^a^ | 48 | 43 | 4 | 1 | Yes^b^ | - | 90% |
| ^1^Hypertrophic cardiomyopathy | 48 | 5 | 34 | 9 | - | No | 71% |
| ^2^Intravascular cardiac pacemakers | 48 | 11 | 32 | 5 | - | No | 67% |
| ^2^Myocardial infarction in the last six months | 48 | 11 | 34 | 3 | - | No | 71% |
| ^1^Cardiac transplantation recipients who develop cardiac valvulopathy | 48 | 25 | 5 | 18 | - | No | 10% |
| ^1^Unrepaired cyanotic heart disease^a^ | 48 | 21 | 15 | 12 | Yes^b^ | - | 44% |
| ^2^Recently placed coronary stents | 48 | 25 | 13 | 10 | - | No | 27% |
| ^1^Atrial septal defect after months of repair^a^ | 48 | 16 | 17 | 15 | Yes^b^ | - | 33% |
| ^2^Ventricular septal defect with repair | 48 | 16 | 17 | 15 | - | No | 35% |
| ^1^Patent ductus arteriosus | 48 | 10 | 21 | 17 | - | No | 44% |
| ^2^Cardiac catheterization without stents | 48 | 7 | 25 | 16 | - | No | 52% |

**Table Four Legend:** The superscript numbers and letters mean the following: ^1^Increased risk of infective endocarditis (IE); **^2^**No increased risk of IE; ^a^Requiring Special Consideration; ^b^use of antibiotics under special considerations. Column six and seven indicate what the FGDP/FDS guidelines recommend: yes = antibiotics recommended; No = antibiotics not recommended. The last column (far right) shows the percentage of participants whose response agreed with the guidelines.
